# Calcineurin is an adaptor required for assembly of the TCR signaling complex

**DOI:** 10.1016/j.celrep.2024.114568

**Published:** 2024-07-31

**Authors:** Shizuka Otsuka, Debjani Dutta, Chuan-Jin Wu, Muhammad S. Alam, Jonathan D. Ashwell

**Affiliations:** 1 Laboratory of Immune Cell Biology, National Cancer Institute, Center for Cancer Research, National Institutes of Health, Bethesda, MD 20892, USA; 2 These authors contributed equally; 3 Lead contact

## Abstract

The serine/threonine phosphatase calcineurin is a component of the T cell receptor (TCR) signalosome, where it promotes T cell activation by dephosphorylating Lck^S59^. Using small interfering RNA (siRNA)-mediated knockdown and CRISPR-Cas9-targeted genetic disruption of the calcineurin A chain α and β isoforms, we find that calcineurin also functions as an adaptor in TCR-signaled human T cells. Unlike inhibition of its phosphatase activity, in the absence of calcineurin A, TCR signaling results in attenuated actin rearrangement, markedly reduced TCR-Lck microcluster formation and recruitment of the adaptor RhoH, and diminished phosphorylation of critical targets downstream of Lck such as TCRζ and ZAP-70. Reconstitution of deficient T cells with either calcineurin Aα or Aβ restores TCR microcluster formation and signaling, as does reconstitution with a phosphatase-inactive Aα chain. These results assign a non-enzymatic adaptor function to calcineurin in the formation and stabilization of a functional TCR signaling complex.

## In brief

Otsuka et al. found that the calcineurin serine-threonine phosphatase has a non-enzymatic adaptor function in activation-induced T cell actin cytoskeletal reorganization, formation of T cell antigen receptor (TCR) microclusters, and the generation of critical proximal signaling events.

## Graphical Abstract

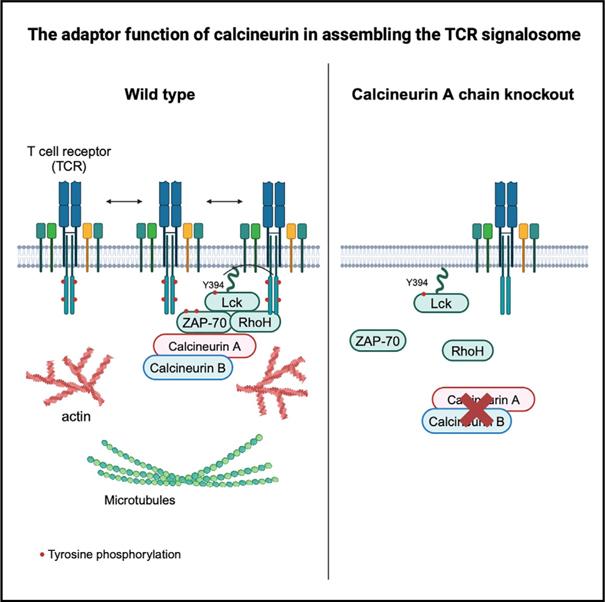

## INTRODUCTION

Activation of T cells via the T cell receptor (TCR) is essential for induction of proliferation and generation of effector functions. Occupancy of the TCR by peptides presented by major histocompatibility complex (MHC) molecules expressed on antigen-presenting cells (APCs) creates minute assemblies of TCR microclusters that eventually coalesce to form the immunological synapse (IS).^1–3^ These microclusters contain a host of enzymes, primarily kinases, and adaptor proteins, molecular scaffolds that lack intrinsic enzymatic activity.^4,5^ The adaptors possess highly specific protein interaction sites that bring together enzymes and their substrates into multi-protein complexes. Among the diverse array of adaptors, there are those with positive (e.g., linker for activation of T cells [LAT], SH2-domain-containing leukocyte protein of 76K [SLP76], GRB2, and Grb2-related adaptor protein [GADS]) and inhibitory (e.g., PAG, SIT, and DOK effects).^6^ Others, such as Wiskott-Aldrich syndrome family protein (WASP), VAV, ADAP, and SKAP-55, have been implicated in the structural organization of the IS, TCR-mediated cell-cell adhesion, and formation of stable T cell-APC conjugates.^7–9^ The concordant binding of enzymes, adaptors, and substrates creates a complex signaling module that propagates signaling distally to initiate biologically important T cell functions.

Cytoskeleton reorganization is a critical feature of the activation of TCR signaling,^7–9^ leading to the phosphorylation of effector molecules. Among them, there are two key adaptor proteins phosphorylated by ZAP-70, LAT and its recruited SLP76, that link the TCR to actin cytoskeletal dynamics. Microtubule repositioning requires the coordinated activation of all key molecules in TCR proximal signaling (Lck, ZAP-70, SLP76, and LAT). The LAT-SLP76-nucleated complexes also recruit several proteins including the adapter molecule NCK and the Rho-family GTPase exchange factor VAV. NCK is constitutively associated with a key regulator of actin cytoskeleton WASP, thus acting as a bridge to recruit WASP to LAT-SLP76 complexes, where WASP is activated by VAV and associates with actin-related protein 2/3 (Arp2/3) to augment actin filament formation. TCR microclusters are subjected to a centripetal force on immobilized stimulatory surfaces driven by retrograde actin flow, which detaches these complexes from the periphery and transports them to the center of the stimulatory surface over time.^10^ The extensive network of actin filaments contributes to not only the generation but also the stabilization of the TCR supramolecular activation clusters (SMACs), with well-demarcated zones such as the peripheral SMAC and the central SMAC.^11^ The retrograde flow of actin filaments is supported by contraction forces of actomyosin II, which coordinates with the cytoskeleton to orchestrate the inward transport of TCR clusters. There is emerging evidence for the role of Rho family GTPases in adhesion and migration via regulation of the cytoskeleton. Classic members of this family include RhoA, Rac1, and CDC42, which shuttle between the active GTP-bound and inactive GDP-free forms to regulate actin assembly and disassembly.^12,13^ An atypical Rho GTPase that lacks catalytic activity, RhoH, has also been reported to regulate IS formation, although whether this was due to an effect on cytoskeletal reorganization is unclear.^14^

Calcineurin is a widely expressed calcium/calmodulin-dependent serine/threonine phosphatase that is activated by increases in intracellular Ca^2+^. In T cells, its major function has been thought to be the dephosphorylation of nuclear factor of activated T cells (NFAT) transcription factors, which allows their translocation into the nucleus and induction of essential cytokines such as interleukin (IL)-2. Calcineurin is a heterodimer containing a 58–59 kDa catalytic A chain having three different isoforms (α, β, and γ) and a 19 kDa regulatory B chain.^15^ Calcineurin Aα and Aβ are ubiquitously expressed, whereas calcineurin γ is restricted to the testis and brain. Although most domains of α and β isoform are much same, the difference is an N-terminal proline-rich region in the β isoform. Despite their high degree of homology, loss of calcineurin Aα and Aβ had differing effects on T cell development and function. Although thymocyte development was normal in calcineurin Aα-deficient mice, antigen-induced T cell activation was impaired.^16^ Mice lacking calcineurin Aβ, on the other hand, had a large reduction in the number of single positive thymocytes and correspondingly fewer peripheral T cells that were hyporesponsive to TCR-mediated activation and prone to undergo apoptosis.^17,18^ This severe phenotype of calcineurin Aβ-deficient T cells is consistent with the quantitative immunoblotting experiments showing calcineurin Aβ as a predominant isoform expressed in murine T cells.^19^

Although increased calcineurin activity in T cells is well known to be critical for the activation of NFATs,^20,21^ we reported that it is also a component of the TCR signalosome and participates in proximal signaling by dephosphorylating Lck^S59^ and allowing normal signal propagation.^22,23^ In initial experiments using small interfering RNA (siRNA) to knock down calcineurin A, we observed that the effect on signaling was qualitatively different from inhibition of calcineurin activity, prompting us to investigate a possible non-enzymatic role for calcineurin in TCR signaling. Here, we show that calcineurin A is required for the initial activation of TCRs and the formation of TCR microclusters, and in its absence, there is reduced recruitment of Lck, ZAP-70, and RhoH to the activated TCR, failure of cytoskeletal and microtubule reorganization, and loss of ζ phosphorylation and subsequent downstream signaling.

## RESULTS

### Knockdown of the calcineurin A subunit has a more profound effect on TCR signaling than inhibition of phosphatase activity

**Figure 1.**
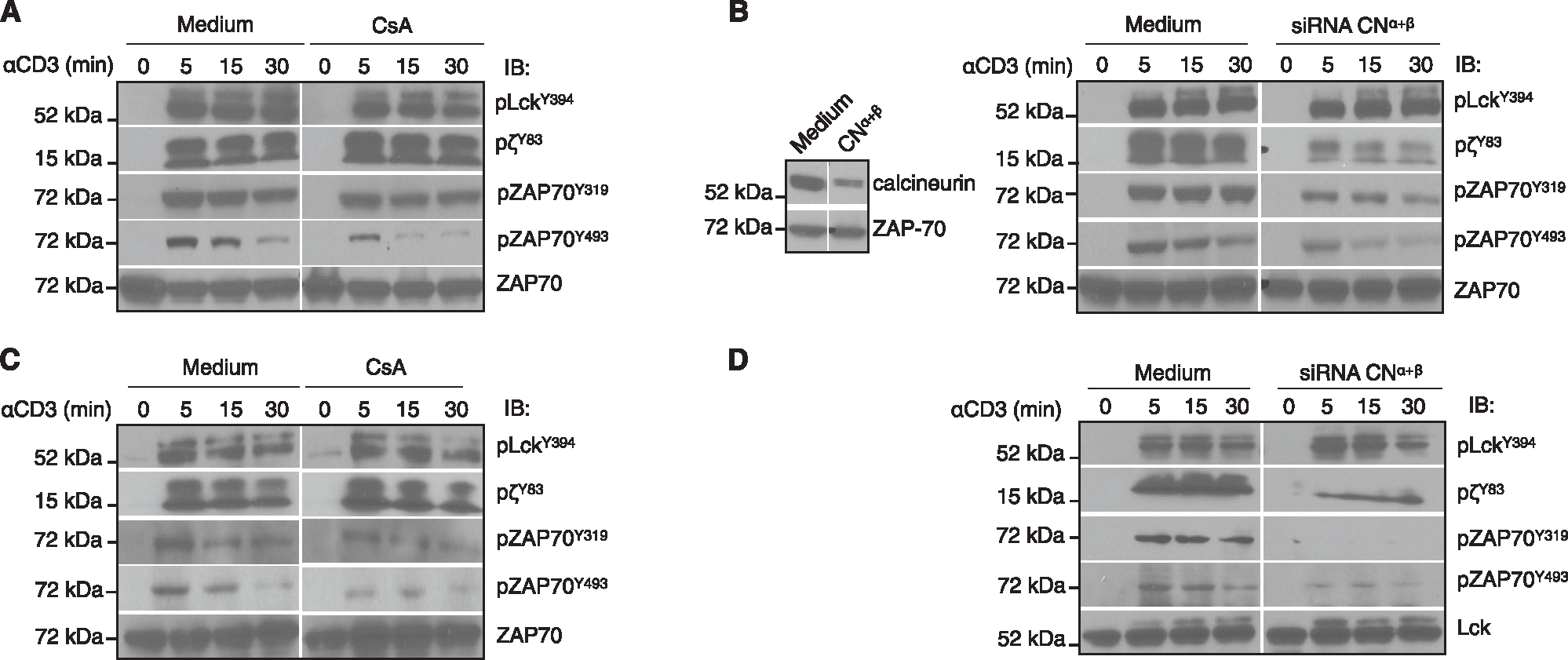
Signaling dichotomy in Jurkat cells treated with CsA/siRNA for calcineurin A (α+β isoforms) Jurkat cells (A and B) and human T cells (C and D) were treated with CsA at 37°C or transfected with the calcineurin A siRNA cocktail (α and β isoforms) and then activated with soluble anti-CD3 and anti-mouse immunoglobulin G (IgG) for the indicated times. Cell lysates were resolved on SDS-PAGE and blotted with phospho-specific antibodies. The blots were stripped and reblotted for total cellular proteins. The data are representative of 3 independent experiments.

Tyrosine phosphorylation of components of the TCR proximal signaling pathway is a prominent early marker of TCR-mediated activation. We have previously shown that pharmacologic inhibition of calcineurin inhibited a subset of T cell Lck-dependent signaling events, starting with phosphorylation of ZAP-70^Y493^.^22^ Notably, phosphorylation of the Lck substrates TCRζ (ζ) and ZAP-70^Y319^, the latter being in the interdomain B region and required to relieve auto-inhibition of the kinase,^24^ was unaffected. As shown in our previous paper^22^ and Figure 1A, in Jurkat T cells, cyclosporin A (CsA) reduced activation-induced phosphorylated ZAP-70^Y493^ (pZAP-70^Y493^) but had no effect on earlier events such as phosphorylation of Lck^Y394^, ζ^Y83^, or ZAP-70^Y319^. In contrast, whereas siRNA-mediated knockdown of the catalytic A chain (isoforms α and β) of calcineurin had no effect on Lck, it inhibited phosphorylation of both ζ^Y83^ and ZAP-70^Y319^ (Figure 1B). Similar results were obtained when the calcineurin A chain was knocked down in primary human T cells, demonstrating that the differences between enzymatic inhibition and loss of the catalytic subunit were not confined to Jurkat cells (Figures 1C and 1D). These results strongly suggested that in addition to dephosphorylating Lck^S59^, calcineurin A also has a non-catalytic role in TCR proximal signaling.

### Defective proximal TCR signal activation and TCR microcluster formation in T cells lacking calcineurin A

**Figure 2.**
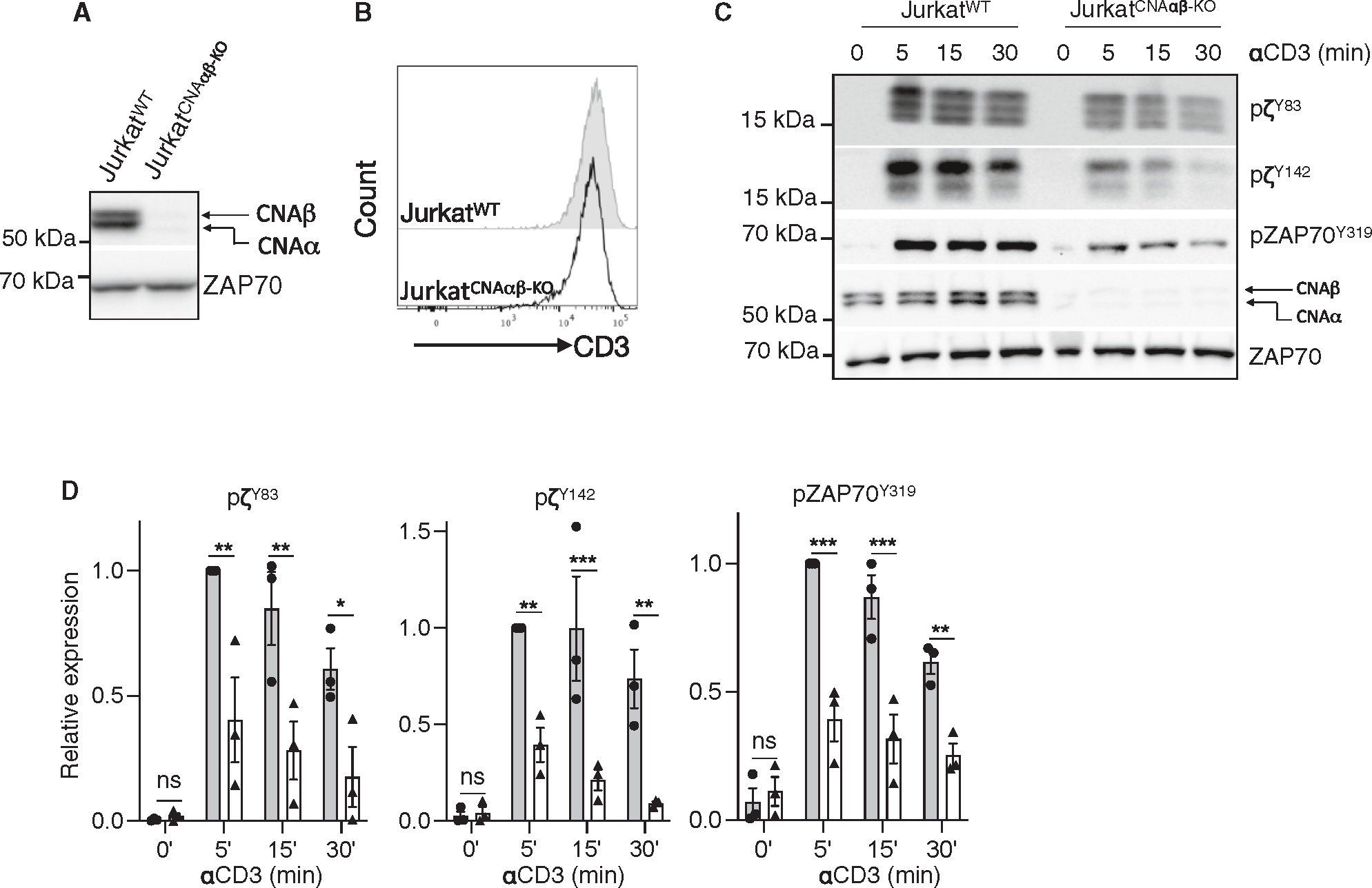
Calcineurin A-deficient cells have a defect in TCRζ-chain phosphorylation and microcluster formation (A) Unstimulated Jurkat^WT^ and Jurkat^CNAαβ-KO^ cells were lysed, and cell lysates were blotted with anti-pan-calcineurin A (calcineurin Aα and Aβ) antibody. (B) CD3ε expression was detected by flow cytometry. (C) Jurkat^WT^ and Jurkat^CNAαβ-KO^ cells were activated with soluble anti-CD3 and anti-mouse IgG, and cell lysates were immunoblotted for pTCRζ (Tyr83 and Tyr142), pZAP-70 (Tyr319), calcineurin A, and ZAP-70. (D) Quantification of the intensity of the indicated bands with ImageJ software. Data are represented as mean ± SEM. Statistical significance was determined by two-way ANOVA with post hoc tests. *n* = 3, *p < 0.05, **p < 0.01, ***p < 0.001, and ns, not significant.

**Figure 3.**
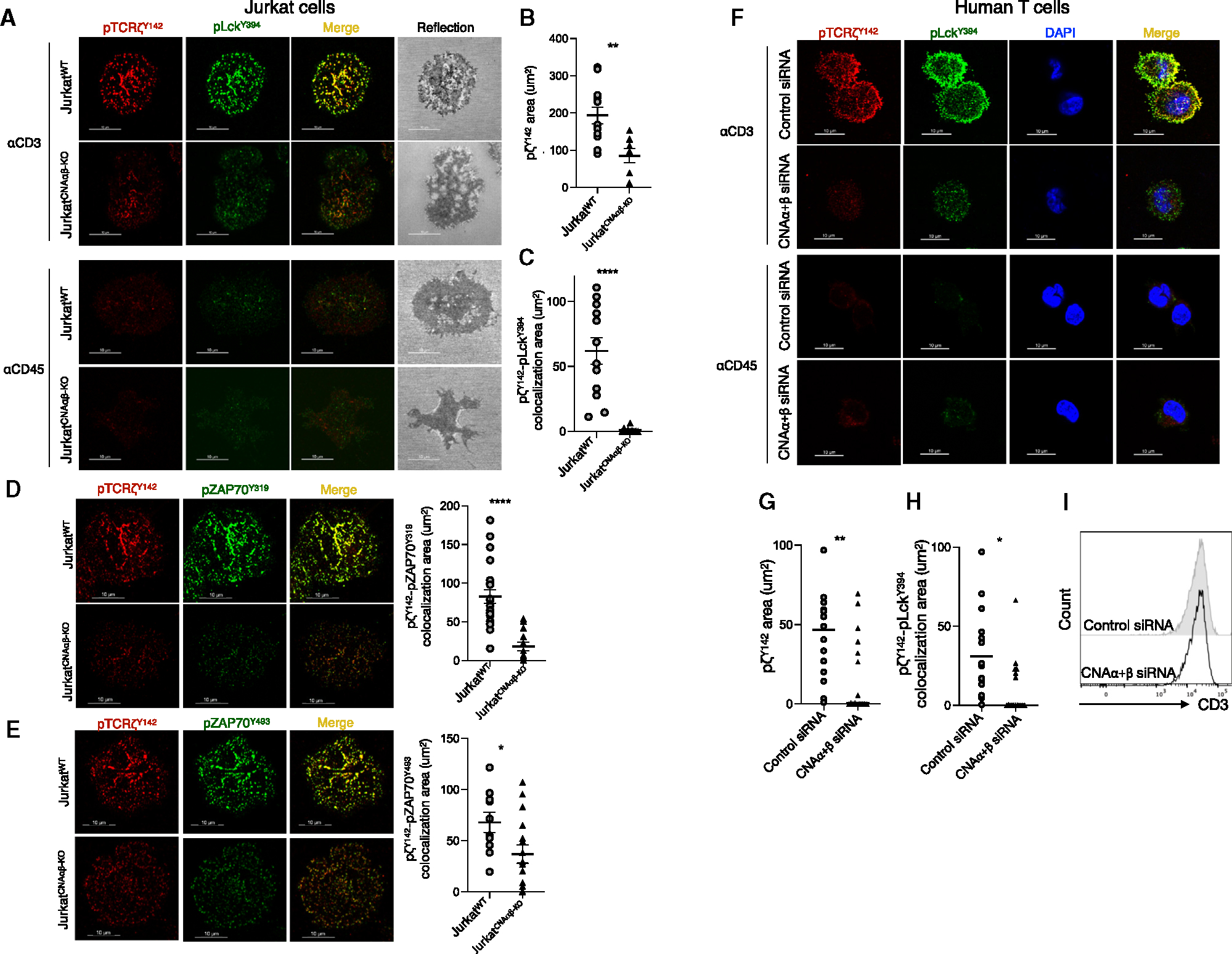
Defective activation-induced TCR microcluster formation in T cells deficient in calcineurin A (A–C) The indicated Jurkat WT and calcineurin A-deficient cells were dropped onto a stimulatory coverslip (coated with anti-CD3) or non-stimulatory coverslip (coated with anti-CD45) for 5 min prior to fixation. pTCRζ^Y142^ is shown in red, pLck^Y394^ is shown in green, and the cell reflection image is shown in gray (scale bar, 10 μm). In each case, a single confocal slice is shown. The pTCRζ^Y142^ cluster area (B) and co-localization area of pLck^Y394^ and pTCRζ^Y142^ (C) in Jurkat^WT^ (*n* = 12) and Jurkat^CNAαβ-KO^ cells (*n* = 10) were analyzed by Imaris imaging software. The data are representative of 2–3 independent experiments. Data are represented as mean ± SEM. Statistical significance was determined by unpaired t test. **p < 0.01 and ****p < 0.0001. (D and E) The indicated cells were dropped onto coverslips and analyzed as in (A). pTCRζ^Y142^ is shown in red and pZAP-70^Y319^ (D) or pZAP-70^Y493^ (E) is shown in green (scale bar, 10 μm). In each case, a single confocal slice is shown. Co-localization areas were analyzed by Imaris imaging software. Jurkat^WT^ (*n* = 22) and Jurkat^CNAαβ-KO^ cells (*n* = 13) in (D) and Jurkat^WT^ (*n* = 10) and Jurkat^CNAαβ-KO^ cells (*n* = 16) in (E) were analyzed. Data are represented as mean ± SEM. Statistical significance was determined by unpaired t test. **p < 0.05 and ****p < 0.0001. (F–H) Primary human T cells treated with control or calcineurin Aα+Aβ siRNA were dropped onto anti-CD3- or anti-CD45-coated coated coverslips for 5 min prior to fixation. pTCRζ^Y142^ is shown in red, pLck^Y394^ is shown in green, and DAPI is shown in blue (scale bar, 10 μm), and in each case, a single confocal slice is shown. The areas of pTCRζ^Y142^ clusters (G) and pLck^Y394^ and pTCRζ^Y142^ co-localization (H) in control (*n* = 16) and calcineurin Aα+Aβ knockdown human primary T cells (*n* = 17) were quantitated by Imaris imaging software. The data are representative of 2 independent experiments. Data are represented as mean ± SEM. Statistical significance was determined by unpaired t test. *p < 0.05 and **p < 0.01. (I) CD3ε expression on human primary T cells transfected with control or calcineurin Aα+Aβ siRNA was analyzed by flow cytometry.

To investigate the molecular mechanism by which the calcineurin A chain affects TCR signaling, we used CRISPR-Cas9 technology to generate Jurkat cell clones lacking both calcineurin Aα and Aβ isoforms (Jurkat^CNAαβ-KO^), which was confirmed by immunoblotting (Figure 2A). Although murine T cells express more calcineurin Aβ than Aα protein,^19^ the reverse appeared to be true for Jurkat cells. Although simple immunoblotting of primary human T cells for calcineurin A did not clearly resolve two discrete bands, siRNA knockdown of *PPP3CA* clearly demonstrated that Aβ was the more abundant species (Figure S1). Although both cell lines had similar levels of TCR and ZAP-70 (Figures 2B and 2C), there was a large reduction in activation-induced phosphorylation of ζ^Y83^, ζ^Y142^, and ZAP-70^Y319^ in Jurkat^CNAαβ-KO^ compared to wild-type (WT) cells (Figures 2C and 2D). Given that Lck-mediated phosphorylation of ζ is the apical event in the proximal signaling pathway, we assessed the assembly of the TCR signaling machinery by immunofluorescence confocal microscopy.^25^ Jurkat cells were dropped onto glass surfaces coated with anti-CD3 (stimulatory) or anti-CD45 (non-stimulatory) and stained for phosphorylated Lck^Y394^ (pLck^Y394^) and phosphorylated ζ^Y142^ (pζ^Y142^). pLck^Y394^ and pζ^Y142^ microclusters formed in the contact area between WT cells and anti-CD3-coated glass but not in the contact area with anti-CD45-coated glass (Figure 3A). In contrast, cells lacking calcineurin A had fewer and smaller microclusters. Furthermore, *en face* images of the WT cells displayed a perfect co-localization of pLck^Y394^ and pζ^Y142^, whereas TCR-mediated activation of Jurkat^CNAαβ-KO^ cells resulted in very little co-localization (Figures 3B and 3C). Lack of calcineurin A also reduced the accumulation of pZAP-70^Y319^ and pZAP-70^Y493^ in the pζ^Y142^-containing microclusters (Figures 3D and 3E). Similarly, while having no effect on cell surface TCR expression, knockdown of calcineurin Aα and Aβ in primary human T cells hampered microcluster formation and co-localization of pLck^Y394^ with pζ^Y142^ (Figures 3F–3I). Taken together, calcineurin A is required at the earliest steps in the initiation of TCR microcluster formation and signaling.

### Calcineurin Aα and Aβ are redundant for TCR signaling

**Figure 4.**
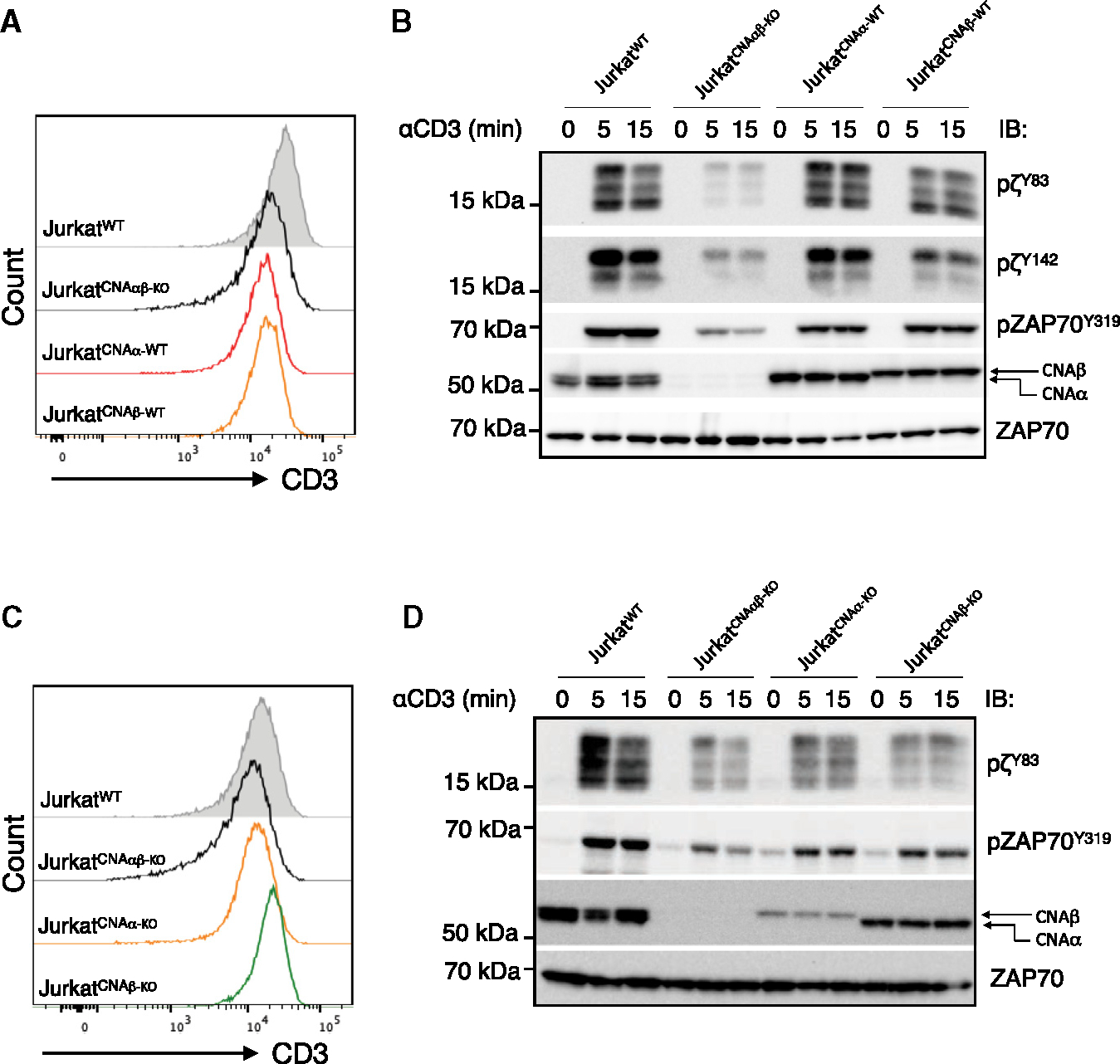
Reconstitution of calcineurin Aα and Aβ restores TCRζ and ZAP-70 phosphorylation (A) Jurkat^CNAαβ-KO^ cells stably expressing WT calcineurin Aα or Aβ were analyzed for cell surface CD3ε expression by flow cytometry. (B) Jurkat^WT^, Jurkat^CNAαβ-KO^, Jurkat^CNAα-WT^, and Jurkat^CNAβ-WT^ cells were activated with soluble α-CD3 and anti-mouse IgG for the indicated times. Cell lysates were immunoblotted with pTCRζ (Tyr83 and Tyr142), pZAP-70 (Tyr319), calcineurin A, and ZAP-70. (C) Jurkat^WT^, Jurkat^CNAαβ-KO^, Jurkat^CNAα-KO^, and Jurkat^CNAβ-KO^ cells were stained with anti-CD3ε antibody and analyzed by flow cytometry. (D) Jurkat^WT^, Jurkat^CNAαβ-KO^, Jurkat^CNAα-KO^, and Jurkat^CNAβ-KO^ cells were activated with soluble anti-CD3 and anti-mouse IgG, and cell lysates were immunoblotted for pTCRζ (Tyr83), pZAP-70 (Tyr319), calcineurin A, and ZAP-70.

To ask if there is a difference between the A isoforms, we introduced each calcineurin A isoform into Jurkat^CNAαβ-KO^ cells, designated Jurkat^CNAα-WT^ and Jurkat^CNAβ-WT^, respectively, and clones expressing homogeneous and similar levels of cell surface CD3ε were chosen for study (Figure 4A). Restoration of either calcineurin Aα or Aβ alone restored TCR-mediated phosphorylation of ζ^Y83^, ζ^Y142^, and ZAP-70^Y319^ (Figure 4B), indicating that the phenotype was not due to CRISPR-Cas9 off-target effects and that the isoforms act redundantly. This was also addressed by comparing Jurkat cells expressing similar levels of TCR in which only Aα (Jurkat^CNAα-KO^) or Aβ (Jurkat^CNAβ-KO^) was knocked out (Figure 4C). Loss of either Aα or Aβ alone reduced the phosphorylation of ζ^Y83^ and, in particular, ZAP-70^Y319^ to levels between those of WT and Aαβ double-knockout cells (Figures 4D and S2). Together, these data support the redundancy of α and β isoforms on TCR signaling.

### Calcineurin A catalytic function is dispensable for activation-induced microcluster formation and TCRζ phosphorylation

**Figure 5.**
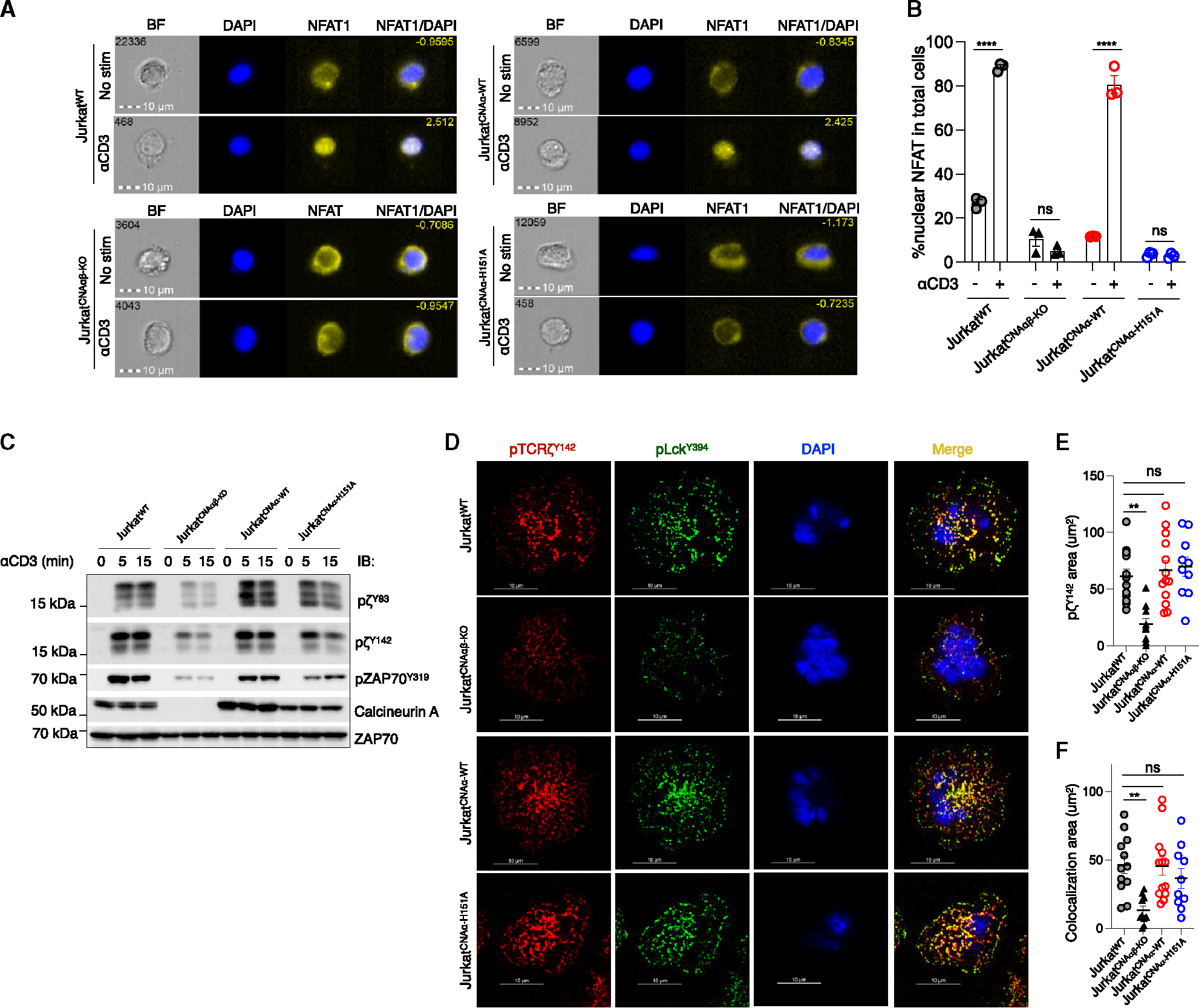
Restoration of TCRζ phosphorylation and microcluster formation by calcineurin A is independent of phosphatase activity (A and B) Jurkat^CNAαβ-KO^ cells were transduced with plasmids encoding WT calcineurin Aα or its inactive mutant (H151A). Images acquired with an ImageStreamX Mark II are representative of unstimulated and anti-CD3-stimulated Jurkat^WT^, Jurkat^CNAαβ-KO^, Jurkat^CNAα-WT^, and Jurkat^CNAα-H151A^ cells after 30 min. DAPI-stained nuclei are shown in blue and NFAT1 is shown in yellow. Quantitative analysis of nuclear translocation was performed with IDEAS image analysis software. Data are represented as mean ± SEM. Statistical significance was determined by two-way ANOVA with post hoc tests. *n* = 3, ****p < 0.0001, and ns, not significant. (C) Jurkat^CNAα-WT^ and Jurkat^CNAα-H151A^ cells were activated with soluble anti-CD3 and anti-mouse IgG for the indicated times. Cell lysates were immunoblotted with the same antibodies as in Figure 2B. (D) Cells were dropped on stimulatory coverslips for 5 min before fixation. Confocal microscopy was performed to study the formation of microclusters. pTCRζ^Y142^ is shown in red and pLck^Y394^ is shown in green. (E and F) Statistical analysis was performed by Imaris imaging software. The pTCRζ^Y142^ cluster area (E) and co-localization area of pLck^Y394^ and pTCRζ^Y142^ (F) were analyzed in Jurkat^WT^ (*n* = 12), Jurkat^CNAαβ-KO^ (*n* = 10), Jurkat^CNAα-WT^ (*n* = 12), and Jurkat^CNAα-H151A^ (*n* = 10) cells. Data are represented as mean ± SEM. Statistical significance was determined by one-way ANOVA with post hoc tests. **p < 0.01 and ns, not significant.

Unlike calcineurin A deficiency, inhibiting calcineurin phosphatase activity with CsA had no effect on ζ phosphorylation or microcluster formation,^22^ suggesting that calcineurin A also functions as an adaptor independently of its enzymatic activity. To confirm this, Jurkat^CNAαβ-KO^ cells were transfected with calcineurin WT Aα (Jurkat^CNAα-WT^) or a catalytically inactive mutant (Jurkat^CNAα-H151A^).^26,27^ To characterize calcineurin activity *in vivo*, reconstituted cells were stimulated with anti-CD3 for 30 min, and calcineurin-dependent NFAT1 nuclear translocation was assessed by imaging flow cytometry (Figure 5A). As expected, 80%–90% of NFAT1 in stimulated Jurkat^WT^ and Jurkat^CNAα-WT^ cells was found in the nucleus. In contrast, there was no NFAT1 nuclear translocation in TCR-stimulated Jurkat^CNAαβ-KO^ or Jurkat^CNAα-H151A^ cells (Figure 5B). Notably, the phosphatase-inactive calcineurin Aα^H151A^ was as effective as WT calcineurin in restoring ζ^Y83^, ζ^Y142^, and ZAP-70^Y319^ phosphorylation (Figure 5C). When examined by confocal imaging, both Jurkat^CNAα-WT^ and Jurkat^CNAα-H151A^ cells responded similarly to stimulation via the TCR as judged by ζ phosphorylation and TCR microcluster formation (Figures 5D and 5E). In addition, both cells displayed perfect co-localization of pLck^Y394^ and pζ^Y142^ (Figure 5F). Therefore, calcineurin A plays a non-catalytic adaptor role in the formation of TCR clusters and proximal signaling.

### F-actin and tubulin aggregation is defective in calcineurin A-deficient cells

**Figure 6.**
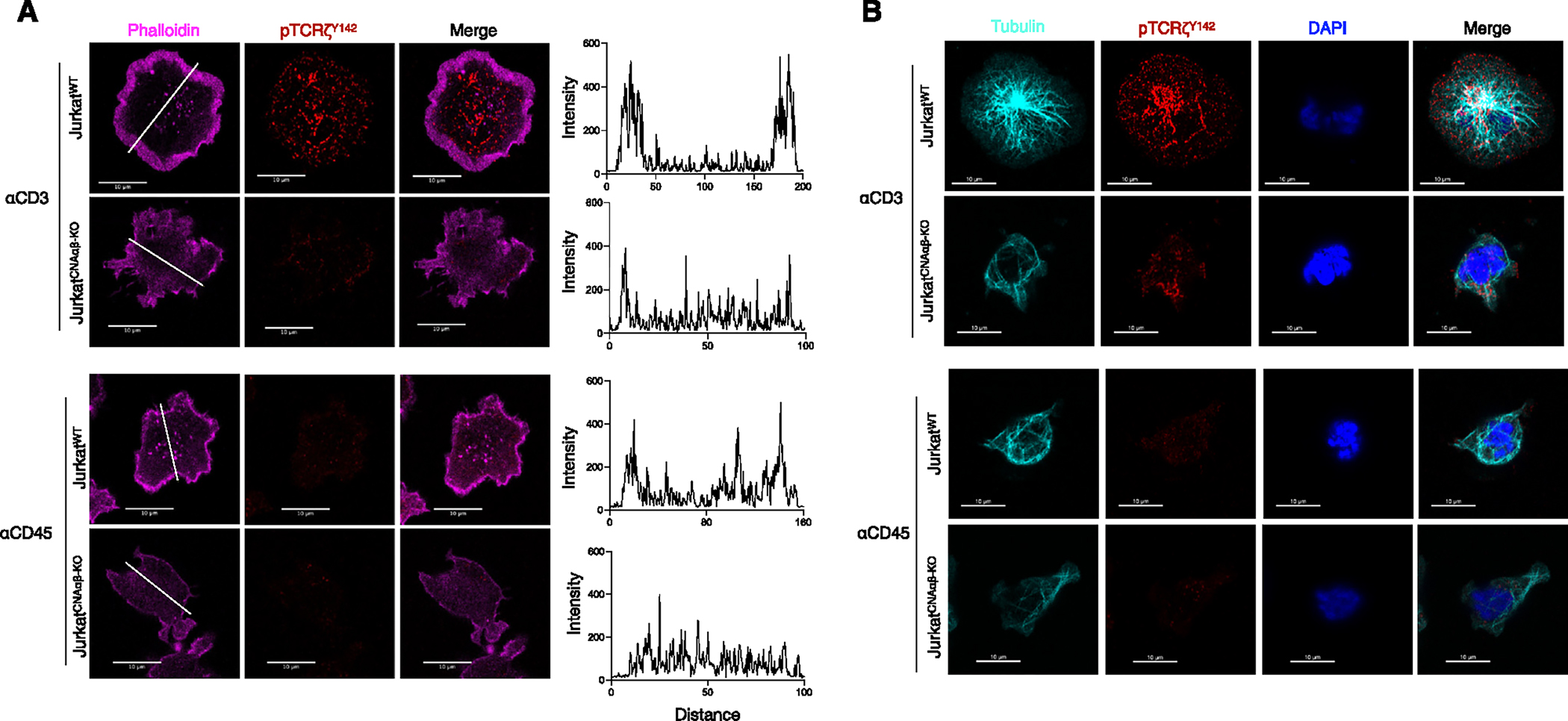
Lack of activation-induced actin polymerization and microtubule reposition in calcineurin A-deficient cells (A) Jurkat^WT^ and Jurkat^CNAαβ-KO^ cells were plated on coverslips coated with anti-CD3 or control anti-CD45. The confocal images were acquired at 5 min after initial contact. pTCRζ^Y142^ is shown in red and phalloidin is shown in purple. In each case, a single confocal slice is shown (scale bar, 10 μm). The data are representative of 2–3 independent experiments. (B) Jurkat^WT^ and Jurkat^CNAαβ-KO^ cells were activated for 5 min as in (A), stained, and visualized by confocal microscopy. Tubulin is shown in cyan, pTCRζ^Y142^ is shown in red, and the DAPI-stained nucleus is shown in blue. The data are representative of 2 independent experiments.

Formation of TCR microclusters following TCR ligation is largely dependent on actin- and microtubule-dependent cytoskeletal reorganization.^28–30^ The TCR is constitutively associated with the underlying actin meshwork, and its engagement induces a dramatic remodeling and segregation of signaling molecules. A first wave of actin remodeling is closely followed by a second phase of microtubule repositioning behind the nucleus. To investigate the role of calcineurin A in TCR-dependent cytoskeletal remodeling, confocal imaging of F-actin structures was performed at the plane of cell contact on a stimulatory coverslip. At 5 min, Jurkat^WT^ cells showed an accumulation of F-actin (stained with phalloidin) throughout the region of coverslip contact, with the appearance of microclusters in the center (Figure 6A). In contrast, Jurkat^CNAαβ-KO^ cells had little F-actin enrichment in the peripheral ring at the interface. Furthermore, inhibition of calcineurin phosphatase activity by CsA had no effect on F-actin accumulation (Figure S3), indicating that cytoskeletal remodeling is independent from its phosphatase activity. We also ask if failure of cytoskeletal reorganization might account for the signaling phenotype in calcineurin A-deficient T cells by performing experiments using cytochalasin D, a cell-permeable inhibitor of actin polymerization. Disruption of actin assembly by cytochalasin D resulted in reduced pLck^Y394^ levels and impaired TCR microcluster formation, similar to that seen with activated Jurkat^CNAαβ-KO^ cells (Figure S4). To estimate the contribution of TCR signaling to microtubule repositioning, Jurkat^WT^ and Jurkat^CNAαβ-KO^ cells were stained with Alexa 594-tagged anti-β-tubulin antibodies. Jurkat^WT^ cells, but not Jurkat^CNAαβ-KO^ cells, had complete repositioning of the microtubule-organizing center (MTOC) toward the center of the cell and away from the cell cortex (Figure 6B). Therefore, the defect in TCR microcluster formation in calcineurin A-deficient cells is due to defective activation-induced actin and microtubule redistribution.

### RhoH recruitment to the TCR is reduced in the absence of calcineurin A

**Figure 7.**
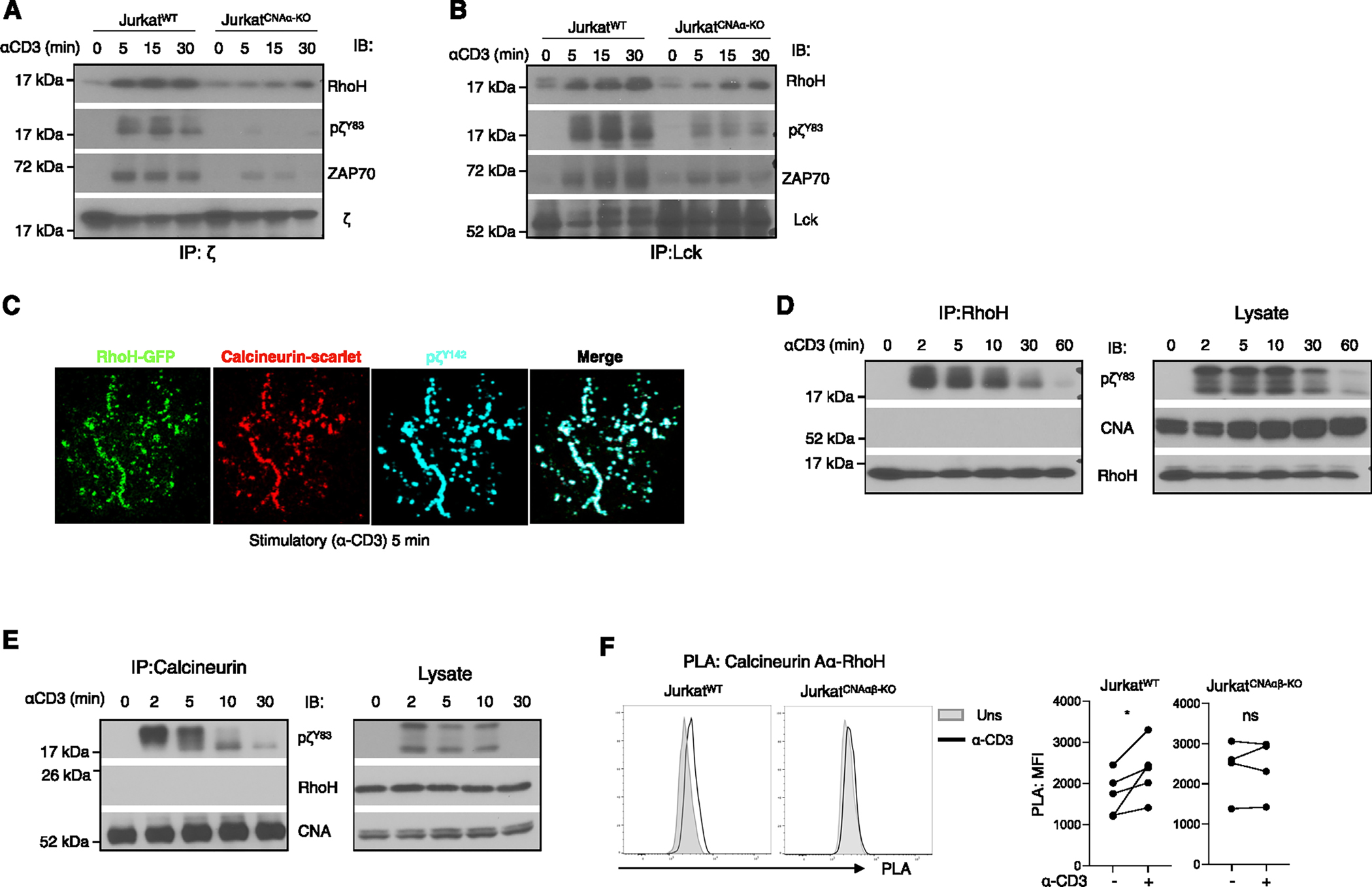
RhoH association with activated TCR molecules is reduced in the absence of calcineurin A (A and B) Jurkat^WT^ and Jurkat^CNAα-KO^ cells were activated for the indicated times and lysed, and the indicated molecules were immunoprecipitated. Washed immunoprecipitates were separated by SDS-PAGE and analyzed for pTCRζ^Y83,^ RhoH, and ZAP-70. Striped membranes were reprobed with antibodies against the precipitated proteins. The data are representative of 2–3 independent experiments. (C) Jurkat cells expressing RhoH-GFP and calcineurin Aα-mScarlet were plated on coverslips coated with anti-CD3 for 5 min. RhoH is shown in green, calcineurin is shown in red, and pTCRζ^Y142^ is shown in cyan. (D and E) Jurkat cells were activated for different time points and subjected to immunoprecipitation using protein G-conjugated anti-calcineurin or anti-RhoH antibody. Interaction of signaling protein pTCRζ^Y83^ was analyzed by immunoblot. Striped membranes were reprobed with anti-pan calcineurin A or anti-RhoH antibody to ensure equal loading of the precipitated complex. Whole-cell lysates from the above were also subjected to immunoblot with the indicated antibody. (F) Jurkat^WT^ (*n* = 5) and Jurkat^CNAαβ-KO^ (*n* = 4) cells are stimulated with plate-coated α-CD3 for 15 min. PLA signals of calcineurin Aα and RhoH interaction were detected by flow cytometry. Statistical significance was determined by paired t test. *p < 0.05 and ns, not significant.

Studies have implicated the hematopoietic-specific GTPase RhoH in the compartmentalization of Lck, ZAP-70, and TCRζ to the IS.^31^ *Rhoh*^−/−^ T cells display impaired TCR-mediated phosphorylation of ITAMs and immune synapse formation but intact Lck kinase activity.^14^ Because of the similarity between the RhoH-deficient and calcineurin A-deficient phenotypes, we explored the possible relationship between these two molecules. As shown by co-immunoprecipitation, RhoH associated with TCRζ and Lck upon activation in WT Jurkat T cells, a response that was markedly decreased in the absence of calcineurin Aα (Figures 7A and 7B). Confocal imaging showed both RhoH and calcineurin co-localized with pζ^Y142^ in microclusters (Figure 6C). To ask if calcineurin A and RhoH directly interact, co-immunoprecipitation with calcineurin A or RhoH was performed. Although anti-RhoH and anti-calcineurin co-immunoprecipitated pζ^Y83^ from TCR-stimulated cells with similar kinetics, neither co-immunoprecipitated the other (Figures 7D and 7E). Proximity ligation assays (PLAs), which permit detection of protein-protein interactions at distances of <40 nm, are more sensitive than co-immunoprecipitation, at least in part because the interactions are detected *in situ* and thus not affected by detergents or washing of beads. Cells were permeabilized, incubated with mouse anti-calcineurin Aα and rabbit anti-RhoH, and, after the PLA was performed, analyzed by flow cytometry. TCR-mediated activation resulted in a small but clear shift of the entire peak in Jurkat^WT^ cells (Figure 7F). In contrast, there was little change in the PLA signal in activated Jurkat^CNAαβ-KO^ cells. Together, the results show that calcineurin A is required for activation-induced RhoH recruitment to TCR microclusters, where they interact weakly or perhaps indirectly with each other.

## DISCUSSION

Calcineurin consists of a catalytic A chain and a regulatory B chain. There are two widely expressed A isoforms, α and β, and the minor isoform γ, which is only expressed in testis and brain.^15,21^ In spite of 85% amino acid identity between the isoforms, they are not redundant with respect to T cell development and function. Calcineurin Aβ knockout mice had reduced numbers of CD4^+^CD8^−^ and CD4^−^CD8^+^ thymocytes and peripheral T cells, the latter having defects in proliferation and IL-2 production when stimulated via the TCR or by PMA and ionomycin, and a large reduction in Bcl-2 levels leading to increased spontaneous apoptosis.^17,18^ Loss of calcineurin Aα, on the other hand, had no obvious effect on thymocyte development but caused a substantial defect in mature T cell antigen-induced activation.^16,32^ Based on quantitative immunoblotting, the β isoform accounts for approximately 75% of the total A chain expressed in mouse T and B cells,^19^ which was thought to account for the more severe immune phenotype in Aβ knockout mice. Interestingly, although real-time qPCR with human T cells detected similar transcript levels,^33^ we found that calcineurin Aα protein is more abundant than Aβ in primary human T and Jurkat cells. The roles of the two A isoforms in TCR proximal signaling have not been investigated. Knockout of either isoform resulted in decreases in TCR-induced phosphorylation, which was restored by reintroduction of either Aα or Aβ in calcineurin A-deficient cells. Therefore, although there may be subtle differences in their efficacy, Aα and Aβ act redundantly as adapters necessary to initiate the assembly of the TCR signalosome.

Membrane-associated adaptors play a pivotal role in coupling proximal TCR signaling molecules to distal events. By definition, such adaptors lack intrinsic enzymatic activity but contain domains that mediate protein-protein interactions.^9^ The Rho family of GTPases, including CDC42 and Rac, is a class of adaptors that function in cytoskeleton-dependent spreading. New imaging technologies and the availability of genetically deficient mice have provided insights into the role of Rho GTPases in the integration of downstream signaling pathways. RhoH, an atypical Rho family member, is a hematopoietic-specific GTPase that binds but cannot hydrolyze GTP to GDP, and unlike classical GTPases, its biological functions are transcriptionally regulated.^34^ The role of RhoH in thymocytes and peripheral TCR signaling is well documented.^35^ Upon TCR activation, RhoH is phosphorylated by kinases such as Lck, which promotes its interaction with ZAP-70 via the immunoreceptor tyrosine-based activation motif (ITAM)-like (pseudo-ITAM) motif of RhoH.^14^ Since RhoH has a CAAX box, a CKIF motif at its C terminus, which facilitates the recruitment of RhoH to the membrane, it works as a shuttle to deliver intercellular ZAP-70 to membrane-associated phosphorylated TCRζ for the initiation of further downstream events. There is a striking phenotypic similarity between the loss of calcineurin A and the loss of RhoH. TCR-activated RhoH-deficient T cells had normal levels of p-Lck^Y394^ but decreased phosphorylation of TCRζ caused by impaired association of Lck with TCRζ and aberrant stable IS formation.^14,31^ Therefore, RhoH regulates localization of Lck as well as ZAP-70 in the IS, but the molecular details of how it does this remain to be elucidated. Our previous experiments using Lck and ZAP-70 inhibitors as well as ZAP-70-deficient (P116) Jurkat cells suggested that Lck-dependent phospho-ZAP-70 recruits calcineurin independently of its own catalytic activity.^22^ The finding that RhoH recruitment to Lck and TCRζ following activation is greatly diminished indicates that the recruitment of calcineurin is upstream of RhoH, and taken together, these observations are consistent with the possibility that calcineurin is an adaptor that facilitates Lck-TCRζ interactions, their accumulation in microclusters, and ZAP-70 phosphorylation. This provides a potential molecular mechanism by which loss of calcineurin leads to the failure of TCRζ phosphorylation and Lck-ζ-ZAP-70 microcluster formation and raises the question of how calcineurin regulates these molecular dynamics. One candidate molecule that might be involved in this mechanism is the 36 kDa adopter protein RACK1 (receptor for activated C kinase 1), which was reported to bind calcineurin.^36^ In T cells, RACK1 preferentially binds to a pool of active p-Lck^Y394^ and the F-actin crosslinking protein α-actinin-1, and this complex is required for the cytoskeletal reorganization that leads to the redistribution of Lck following TCR ligation.^37^ These observations raise the possibility that a calcineurin A-RACK1 complex may form a stable association between active p-Lck^Y394^ and TCRζ to regulate the dynamics of microcluster formation.

In general, the dynamic actin architecture is regulated by its assembly and disassembly during cellular processes such as migration, adhesion/cell-cell interactions, and activation.^38^ Several proteins, identified as actin nucleators, initiate actin chain assembly from monomers into branched actin filament networks. The first major actin nucleator is an Arp2/3 complex, which is highly conserved in eukaryotic cells. The Arp2/3 complex is activated by nucleation-promoting factors including WASP and WASP-family verprolin-homologous protein (WAVE), which recruit 1–3 actin subunits and promote a conformational change within the Arp2/3 complex.^39^ Actin regulation is also necessary for optimal TCR triggering, microcluster formation, and generation of a stable IS. Inhibitors of cytoskeletal reorganization such as jasplakinolide and cytochalasin D not only prevent TCR signaling but also inhibit TCR-induced spreading on anti-TCR-coated plates.^28,29,40^ In addition, the rapid polymerization of actin filaments is loosely linked to microtubule repositioning, which creates an organized junction between the T cell and the APC for ongoing activation, with the MTOC interposed between the nucleus and the IS.^41^ Some of the molecular machinery that orchestrate actin networks in T cells overlap with general actin regulation. For example, T cells lacking Arp2/3 have decreased cell surface TCR levels, T cell-APC conjugation, and IS formation.^42^ Ablation of WASP in T cells resulted in abnormally smooth cell membranes and defective proliferation, actin polymerization, and the assembly of IS.^43^ Calcineurin is an important regulator of cytoskeletal and morphological integrity in neurites, myocytes, and kidney cells.^44,45^ A number of calcineurin substrates, such as microtubule-associated protein 2 and tau factor, are located within the cytoskeleton^46,47^ and act as crosslinkers between the microtubule and actin networks. Furthermore, the microtubule-binding protein dynamin I in neurons and the actin-binding protein synaptopodin in kidneys are also calcineurin substrates.^48,49^ Our experiments, however, demonstrate that TCR-activation-induced cytoskeletal regulation by calcineurin A is independent of its phosphatase activity and is unlike many of the examples identified in other cell types. Previous studies using microscopy techniques provided direct evidence for membrane cytoskeleton co-localization of the A-kinase-anchoring protein 79 (AKAP79)-calcineurin complex in association with disc large 1 (Dlg1), a member of the membrane-associated guanylate kinase protein family (MAGUK), in neurons.^50^ In T cells, Dlg1 is known to associate with signaling- and cytoskeleton-related molecules important for T cell activation and polarity. It forms complexes with Lck-ZAP-70-WASP in the membrane and enhances actin polymerization following TCR/CD28 engagements.^51^ Similar to our calcineurin-deficient cells, Dlg1 silencing disrupted not only microcluster formation but also microtubule network organization at the synapse.^30^ Taken together, calcineurin and AKAP79 may form a complex with Dlg1 and have a role in regulating TCR-induced Dlg1-WASP-mediated actin polymerization and MTOC repositioning.

Calcineurin is an essential signaling component for T cell activation due to its participation in activation of NFATs.^52^ Inhibition of calcineurin with drugs like CsA and FK506 is a staple of immunosuppressive therapies.^53^ While its role in NFAT inhibition is well characterized, studies from our laboratory found that it was rapidly recruited to the TCR signalosome, where its phosphatase activity counters an inhibitory phosphorylation of Lck at Ser-59 and promotes T cell adhesion to APCs by increasing the affinity of LFA-1 for ICAM1,^22^ adding an NFAT-independent important step in T cell activation. In the context of proximal TCR signaling, inhibition of calcineurin phosphatase activity by pharmacological inhibitors reduced phosphorylation of ZAP-70^Y493^ and its downstream substrates but not upstream events such as phosphorylation of TCRζ and ZAP-70^Y319^.^22^ The importance of T cell pLck^S59^ as a calcineurin target was underlined by the finding that although NFAT was markedly inhibited, CsA was ineffective in acute graft-versus-host disease when T cells expressed non-phosphorylatable Lck^S59A^. ^23^ The data in this report demonstrate that calcineurin has yet another, non-catalytic role in TCR signaling. In its absence, there is a failure to assemble the signaling machinery into microclusters, which are necessary to propagate and amplify downstream signaling cascades. Calcineurin, therefore, joins the list of TCR signaling scaffold proteins and, given its essential role in TCRζ phosphorylation, participates in the very earliest events.

### Limitations of the study

Although we identified calcineurin as an essential apical adaptor in forming the TCR signalosome, a detailed molecular mechanism remains unclear. PLA experiments provided evidence that calcineurin and RhoH are in very close proximity in microclusters, but whether this interaction is direct or mediated via intermediary molecular interactions is unknown. Another consideration is that TCR-mediated activation was initiated by stimulation with anti-CD3 antibodies. Although widely used in such studies, there may be subtle but important differences between this and physiological stimulation with a cognate peptide presented by MHC molecules on the surface of APCs. Similarly, by using TCR crosslinking antibodies to initiate signaling, one cannot assess a possible role for the CD4 and CD8 co-receptors in calcineurin-dependent microcluster assembly.

## STAR★METHODS

### RESOURCE AVAILABILITY

#### Lead contact

Further information and requests for resources and reagents should be directed to and will be fulfilled by the lead contact, Jonathan D. Ashwell (jda@pop.nci.nih.gov).

#### Materials availability

All unique reagents generated in this study are available from the lead contact upon request.

#### Data and code availability

- All data reported in this paper will be shared by the lead contact upon request.
- This paper does not report original code.
- Any additional information required to analyze the data reported in this paper is available from the lead contact upon request.

### EXPERIMENTAL MODEL AND STUDY PARTICIPANT DETAILS

#### siRNA knockdown in Jurkat cells

Five million Jurkat cells were resuspended in 500 μL of antibiotic-free RPMI medium with 10 μL (100 pmol) each of the validated siRNA’s (PP2B-Aα and PP2B-Aβ) and the control siRNA-A, and placed inside a cuvette (Bio-Rad). The cuvettes were pulsed using the BTX electroporator (300V, 10ms, 960μF) and cells transferred into RPMI medium containing 10% fetal calf serum (FCS). Cells were analyzed for calcineurin knockdown after 72 h by immunoblot. At the protein level the extent of calcineurin knockdown was estimated by densitometric analysis using ImageJ software.

#### Plasmids

Two single guiding RNAs (sgRNAs) targeting calcineurin Aα were cloning into pLentiGuide-Puro (Addgene #52963), and two sgRNAs targeting calcineurin Aβ were cloned into pLentiCRISPRv2-neo (Addgene #98292) plasmid using BsmBI restriction sites, as outlined in the protocol from Feng Zhang’s lab (available at Addgene). Calcineurin Aα cDNA was cloned into pMRX-IRESThy1.1 retroviral plasmid^57^ at BamHI and XhoI sites, and calcineurin Aβ cDNA was cloned into pMRX-IRESThy1.1 at BglII and EcoRI sites. For the reconstitution of calcineurin into calcineurin KO cells, sgRNA targeting sequences and protospacer adjacent motif (PAM) sequences were site-mutated to prevent Cas9 targeting while preserving protein amino acid sequence. In addition, calcineurin Aα H151A mutation was introduced via site-directed mutagenesis to disable their phosphatase activities. Site-directed mutagenesis was performed using QuikChange XL kit (Agilent Technologies) according to the manufacturer’s instructions.

#### Calcineurin knockout cell-line generation

Single or double genetic deletions of calcineurin Aα and Aβ were achieved using transduction of CRISPR/Cas9 targeting lentiviral plasmids. One to one mixture of two pLentiGuide-Puro plasmids containing individual sgRNAs targeting calcineurin Aα were transfected with pVSVg, and psPAX2 into HEK293T cells. Jurkat cells were infected with lentiviral supernatants and expanded for 48 h before 1 week of 1 μg/ml puromycin selection. The calcineurin Aα sgRNAs-transduced and puromycin-selected cells were then transduced with 1:1 mixture of two pLentiCRISPRv2-neo plasmids containing individual sgRNAs targeting calcineurin Aβ and selected with 500 μg/ml geneticin G418 for 10 days. These transduced cells were cloned using limited dilution and screened for calcineurin Aα and Aβ single knockout and double knockout cells using immunoblotting.

#### Reconstitution of calcineurin A expression into Jurkat^CNAαβ-KO^ cells

The pMRX-IRESThy1.1 plasmid containing calcineurin Aα, Aα-H151A mutant or Aβ cDNA in which the gRNA targeting sequences were mutated to avoid Cas9-based editing or the empty vector was transfected into Jurkat^CNAαβ-KO^ cells via electroporation at 260 V for 20 s using Gene Pulser Xcell (Bio-Rad Laboratories, Hercules, CA). After culturing for one week, the transfected cells were stained with PE-*anti*-Thy1.1 and sorted for Thy1.1-positive cells using FACSAria (BD Bioscience). Protein levels of the reintroduced calcineurin A were determined by immunoblotting.

#### Plasmid transfection of Jurkat cells

Calcineurin Aα cDNA from pET15b CnA CnB (Addgene# 11787) was cloned into pmScarlet-C1 vector. Cells were suspended in 500 μL of antibiotic-free RPMI medium with 10 μg GFP-RhoH (Addgene #23230) and pmScarlet-C1 containing calcineurin Aα, and placed inside a cuvette (Bio-Rad). The cuvettes were pulsed using the BTX electroporator (300V, 10ms, 960μF) and cells transferred into RPMI medium containing 10% FCS. Confocal imaging of these cells was performed within 72 h.

### METHOD DETAILS

#### Flow cytometry

Cells were washed once in cold PBS containing 0.5% BSA and 0.1% NaN3 (FACS buffer). Surface staining was performed with the anti-CD3ε antibody (HIT3a) for 30 min at 4°C in FACS buffer. Cells were acquired with an LSRII (BD Biosciences) and analyzed with FlowJo software. For image flow cytometry, cells were stimulated with anti-CD3 cross-linked with anti-hamster IgG for 30 min, then permeabilized and fixed with Foxp3/Transcription Factor Staining Buffer Set (eBioscience). DAPI was added and cells acquired with an ImageStreamX Mark II (Amnis). Quantitative analysis of nuclear translocation of NFAT1 was performed with IDEAS image-analysis software (ImageStream/Amnis). NFAT nuclear translocation is expressed as the precent of cells that exhibit a >1 similarity morphology score. Amnis IDEAS software calculates the similarity feature as a pixel-by-pixel correlation between the channel containing the NFAT1 image and the DAPI channel with the nuclear image, and is expressed as the log-transformed Parson’s Correlation Coefficient.

#### Activation of Jurkat cells or human CD4 T cells and immunoblotting

Jurkat cells were incubated with soluble anti-human CD3 (OKT3) for 15 min on ice. In some cases, cells were treated with CsA (100–200 ng/mL) for 2 h at 37°C before the OKT3 incubation. OKT3 was then cross-linked with anti-mouse IgG for the indicated times and the cells lysed in 1% Triton X-100 lysis buffer (150 mM NaCl, 20 mM Tris pH 7.5, 1% Triton X-100) supplemented with protease and phosphatase inhibitors (Roche) for 30 min on ice and centrifuged for 20 min at 13000 rpm. The supernatants were boiled in SDS sample buffer for 10 min. Samples were subjected to SDS-PAGE and transferred to nitrocellulose membranes (*Trans*- Blot Turbo, Bio-Rad). The membranes were incubated for 60 min in 2% skim milk powder in Tris-buffered saline (TBS) with 0.1% Tween (TBS-T) and then incubated overnight at 4°C with the indicated primary antibodies. Membranes were then washed with TBS-T and incubated with the appropriate HRP-conjugated anti-rabbit or mouse Ig secondary antibodies for 1 h at room temperature. Immunodetection was performed by enhanced chemiluminescence (SuperSignal West Femto or SuperSignal West Dura). Human CD4^+^ T cells were isolated from buffy coats of healthy volunteers (NIH Blood Bank) using the Human CD4 cell recovery column kit (Cedarlane), according to the manufacturer’s instructions. Human CD4 T cells were stimulated as above. In some cases, membranes were stripped with stripping buffer for 10min and reblotted for the total proteins. SDS sample buffer was purchased from Quality Biologicals Inc, and Restore PLUS Western blot Stripping Buffer from Thermo Scientific.

#### Immunoprecipitation

T cells were lysed in ice-cold lysis buffer (150 mM NaCl, 20 mM Tris pH 7.5, 1% Triton X-100 supplemented with protease inhibitor and phosphatase inhibitor; Roche). Lysates were cleared by centrifugation at 13000 rpm for 20 min and subjected to immunoprecipitation using agarose-conjugated anti-TCRζ and anti-Lck, or Protein G beads incubated with anti-calcineurin Aα, anti-RhoH or an IgG1 control antibody for 4–6 h. The beads were washed in ice-cold lysis buffer and eluted by boiling in SDS sample buffer and resolved by SDS-PAGE.

#### Confocal microscopy and image analysis

Cells were allowed to spread on coverslips as described (Bunnell et al., 2003). In brief, cells were dropped onto poly-lysine-treated four-chambered glass coverslips coated with stimulatory anti-CD3 (UCHT-1) or non-stimulatory anti-CD45 (H130) antibody at 10 μg/mL. In some cases, cells were pretreated with cytochalasin D (10 μM) or CsA (100 ng/mL) before being dropped onto coverslips. Cells were resuspended in warm medium without phenol red supplemented with 25 mM HEPES, pH 7, and dropped at the bottom of the chamber followed by incubation at 37°C for 5 min. The cells were fixed with 2.4% paraformaldehyde for 30 min and permeabilized with Triton X-100 for 5 min. The slides were blocked in blocking buffer (2% goat serum) for 30 min and incubated with primary antibodies for 60 min at the appropriate dilution (anti-Lck (Y394), anti-CD247 (ζ, Y142), anti-ZAP-70 (Y319), anti-ZAP-70 (Y493), 1:500). Cells were washed and stained with labeled secondary antibodies, Alexa Fluor 568 anti-mouse IgG (1:1000), Alexa Fluor 488 anti-rabbit IgG (1:1000) or Alexa Fluor 647 anti-mouse IgG (1:500). For imaging analysis of cytoskeletal organization, cells are stained with phalloidin (Alxa-594 or Alexa 488) or anti-β-tubulin (1:100) for 60 min at room temperature. Images were captured using a Zeiss LSM 780 (ZEISS Research Microscopy Solutions) or Leica Stellaris 8 FLIM (Leica Microsystems Inc) microscope using a 63X, 1.4 NA objective and processed in Imaris software.

#### Immunoprecipitation

T cells were lysed in ice-cold lysis buffer (150 mM NaCl, 20 mM Tris pH 7.5, 1% Triton X-100 supplemented with protease inhibitor and phosphatase inhibitor; Roche). Lysates were cleared by centrifugation at 13000 rpm for 20 min and subjected to immunoprecipitation using agarose-conjugated anti-TCRζ and anti-Lck, or Protein G beads incubated with anti-calcineurin or an IgG1 control antibody for 4–6 h. The beads were washed in ice-cold lysis buffer and eluted by boiling in SDS sample buffer and resolved by SDS-PAGE.

#### *In situ* PLA

Duolink *in situ* PLA enables detection, visualization, and quantification of protein interactions (<40 nm) by flow cytometry. Interaction between calcineurin Aα and RhoH was detected in Jurkat cells by PLA using Duolink *in Situ* detection reagents Red (Sigma) according to the manufacturer’s protocol with minor modifications. In brief, for flow cytometry analysis, cells were stimulated with plate coated anti-CD3 (OKT3) for indicated time, fixed with 4% paraformaldehyde (PFA), permeabilized with 0.2% Triton X- in PBS, and stained with mouse anti-calcineurin Aα and rabbit anti-RhoH primary antibodies in 15 mL tube. Cells were treated with anti-mouse MINUS and anti-rabbit PLUS probes, ligated, and amplified, and the detection was performed by an LSRII (BD Biosciences) and analyzed with FlowJo software.

### QUANTIFICATION AND STATISTICAL ANALYSIS

#### Immunoblot band intensity quantification

Band density was quantified by ImageJ software. A rectangle was placed around each band using the program tools. The gray value (intensity) of each rectangle was obtained as arbitrary area values. The calculated density of each band was normalized to that of ZAP-70 in the same lane.

#### Statistical analysis

All values are presented as mean ± SEM or SD, as indicated. Statistical analysis was performed with GraphPad Prism 10 software. Statistical significance was determined with the unpaired or paired t test and multiple comparisons with two-way or one-way Anova with post hoc tests, and asterisks indicate the level of significant differences *p < 0.05, **p < 0.01, ***p < 0.001, ****p < 0.0001.

## Supplementary Material

1

## Figures and Tables

**KEY RESOURCES TABLE:** 

REAGENT or RESOURCE	SOURCE	IDENTIFIER

Antibodies		
Phospho-Zap-70 (Tyr319)/Syk (Tyr352) Antibody	Cell Signaling Technologies	Cat# 2701; RRID:AB_331600
Phospho-Zap-70 (Tyr493)/Syk (Tyr526) Antibody	Cell Signaling Technologies	Cat# 2704; RRID:AB_2217457
Zap-70 Rabbit Antibody, Clone 99F2	Cell Signaling Technologies	Cat# 2705; RRID:AB_2273231
Phospho-Src Family (Tyr416) Antibody	Cell Signaling Technologies	Cat# 2101; RRID:AB_331697
Pan-Calcineurin A Antibody	Cell Signaling Technologies	Cat# 2614S; RRID:AB_2168458
Anti-NFAT1 monoclonal Antibody, PE, Clone D43B1	Cell Signaling Technologies	Cat# 14335; RRID:AB_2798454
β-Tubulin (9F3) Rabbit mAb (Alexa Fluor 647 Conjugate)	Cell Signaling Technologies	Cat# 3624; RRID:AB_10694204
Phospho-CD3ζ (Y83) Antibody	abcam	Cat# ab68236; RRID:AB_11155460
Phosho-Lck (Y394) Antibody	Santa Cruz	Cat# sc-101728; RRID:AB_1125930
CD3ζ (6B10.2) Antibody	Santa Cruz	Cat# sc-1239; RRID:AB_627020
Lck (3A5) Antibody	Santa Cruz	Cat# sc-433; RRID:AB_627880
Calcineurin A (α-subunit) Antibody	Millipore Sigma	Cat# C1956; RRID:AB_258774
Phosho-CD247 (Y142) Antibody	BD Biosciences	Cat# 558402; RRID:AB_647307
Purified mouse anti-Human CD3 Antibody (UCHT-1)	BD Biosciences	Cat# 555329; RRID:AB_395736
Mouse Anti-CD45 Monoclonal Antibody	BD Biosciences	Cat# 555480; RRID:AB_395872
CD3 Monoclonal Antibody (OKT3)	eBioscience	Cat# 16–0037-85; RRID:AB_468855
PE conjugated anti-CD3 monoclonal antibody (HIT3a)	eBioscience	Cat# 12–0039-42; RRID:AB_10853029
Donkey Anti-Mouse IgG (H + L)	Jackson Immunoresearch	Cat# 715–005-151; RRID:AB_2340759
ECL Mouse IgG, HRP-linked F(ab’)_2_ fragment	Cytiva	Cat# NA9310; RRID:AB_772193
ECL Rabbit IgG, HRP-linked F(ab’)_2_ fragment	Cytiva	Cat# NA9340; RRID:AB_772191
RhoH polyclonal antibody	Thermo Fisher Scientific	Cat#PA5–56443; RRID:AB_2646538
Goat anti-Rabbit IgG Secondary Antibody, Alexa Fluor 488	Thermo Fisher Scientific	Cat# A-11034; RRID:AB_2576217
Goat anti-Mouse IgG1 Secondary Antibody, Alexa Fluor 546	Thermo Fisher Scientific	Cat# A-21123; RRID:AB_2535765
Goat anti-Mouse IgG2b Secondary Antibody, Alexa Fluor 647	Thermo Fisher Scientific	Cat# A-21242; RRID:AB_2535811
Monoclonal anti-RhoH clone 3D3	Sigma-Aldrich	Cat# WH0000399M3; RRID:AB_1843345
Chemicals, peptides, and recombinant proteins		
cOmplete^™^, Mini Protease Inhibitor Cocktail	Roche	Cat# 11836153001
PhosSTOP, Phosphatase inhibitors	Roche	Cat# 4906845001
Cyclosporin A from *Tolypocladium inflatum*	Sigma-Aldrich	Cat# C3662
Cytochalasin D	Sigma-Aldrich	Cat# 250255-M
Alexa Fluor 594 Phalloidin	Thermo Fisher Scientific	Cat# A12381
Alexa Fluor 488 Phalloidin	Thermo Fisher Scientific	Cat# A12379
Critical commercial assays		
SuperSignal^™^ West Femto Maximum Sensitivity Substrate	Thermo Fisher Scientific	Cat# 34096
QuikChange XL kit	Agilent Technologies	Cat#200521
Human CD4 Cell Recovery Kit	Cledarlane	Cat#CL110
Duolink *In Situ* PLA Probe Anti-Rabbit PLUS	Millipore Sigma	Cat# DUO92002
Duolink *In Situ* PLA Probe Anti-Mouse MINUS	Millipore Sigma	Cat# DUO92004
Duolink *In Situ* Detection Reagents Red	Millipore Sigma	Cat# DUO92008
Recombinant DNA		
pLentiGuide-Puro	Sanjana et al.^54^	Addgene, Cat# 52963; RRID: Addgene_52963
pLentiCRISPRv2-neo	Stringer et al.^55^	Addgene, Cat# 98292; RRID:Addgene_98292
GFP-RhoH	Roberts et al.^56^	Addgene, Cat# 23230; RRID:Addgene_23230
pET15b CnA CnB	Mondragon et al.^26^	Addgene, Cat# 11787; RRID:Addgene_11787
mScarlet-C1 vector	Provided by Lawrence E. Samelson	N/A
Experimental models: Cell lines		
Jurkat cells	Provided by Lawrence E. Samelson	N/A
Oligonucleotides		
Control siRNA-A	Santa Cruz	Cat# sc-37007
PP2B-Aα siRNA	Santa Cruz	Cat# sc-36304
PP2B-Aβ siRNA	Santa Cruz	Cat# sc-39195
Software and algorithms		
BD FACSDiva software	BD Biosciences	RRID: SCR_001456
FlowJo	BD Biosciences	RRID:SCR_008520
Imaris	Bitplane	RRID:SCR_007370
GraphPad Prism 10	GraphPad Software	RRID:SCR_002798
ImageStreamX Mark II	Luminex Amnis	RRID:SCR_018589
IDEAS	Luminex Amnis	N/A
BioRender	BioRender	https://www.biorender.com

## Highlights

- Calcineurin is an essential adaptor in activation-induced TCR microcluster formation
- Calcineurin’s phosphatase activity is dispensable for its adaptor function
- Early TCR-dependent actin reorganization depends on the presence of calcineurin
- Calcineurin interacts with another important adaptor, RhoH, in TCR microclusters
