# Supplementary material for: Calcineurin is an adaptor required for assembly of the TCR signaling complex
Source: Cell Rep. Author manuscript; Available in PMC 2024 Sep 17. (PMC11407306; doi:10.1016/j.celrep.2024.114568)
Supplement: 1 [file NIHMS2019532-supplement-1.pdf]

Cell Reports, Volume *43*

Supplemental information

Calcineurin is an adaptor required for assembly of the TCR signaling complex

Shizuka Otsuka, Debjani Dutta, Chuan-Jin Wu, Muhammad S. Alam, and Jonathan D. Ashwell

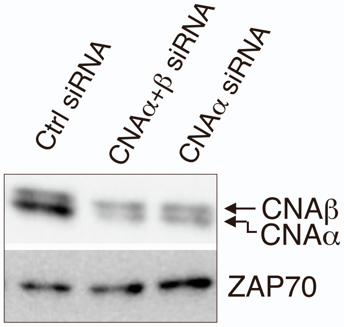

Figure. S1 The protein expression of calcineurin Aα and β in human primary T cells
Control, calcineurin Aα+Aβ, and calcineurin Aα knockdown primary T cell lysates were resolved on SDS and blotted for calcineurin A and ZAP70.

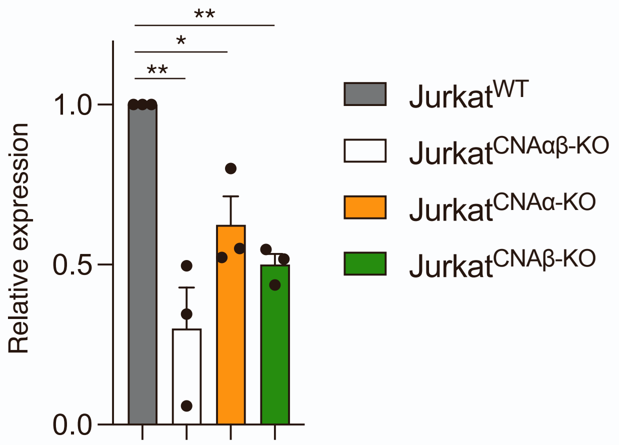

Figure. S2 Quantification of pZAP-70^{Y319} in the indicated Jurkat WT and calcineurin A knock-out cells
The graph shows the relative expression of pZAP-70^{Y319} in the immunoblot from Fig. 4D and two other independent experiments. Data are represented as mean±SEM. n = 3, *P < 0.05, **P < 0.01, ns= not significant.

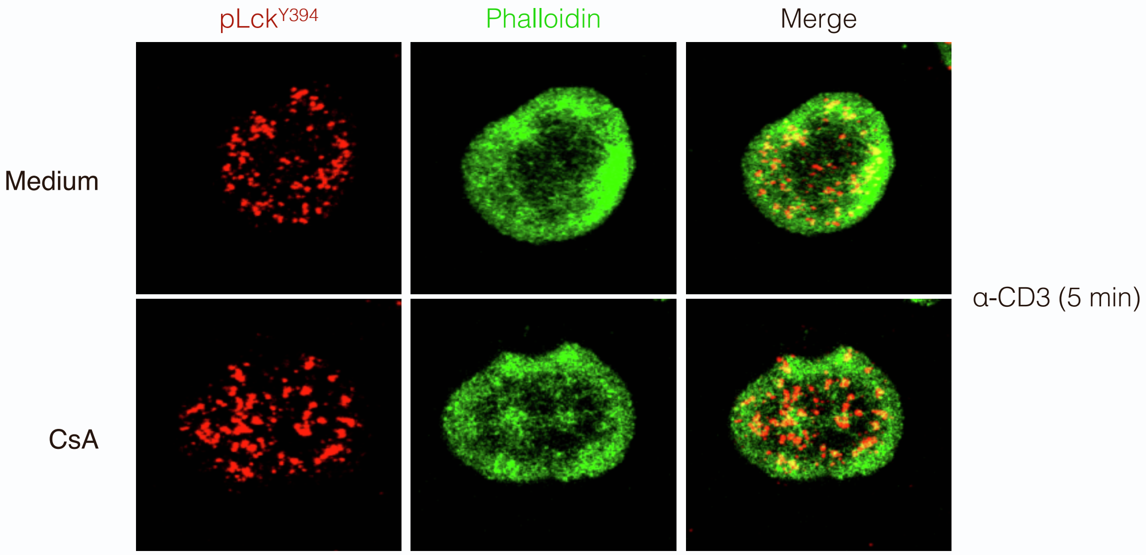

Figure. S3 The phosphatase activity of calcineurin is not required for activation-induced actin accumulation.
Jurkat WT cells were treated with or without CsA and dropped onto a coverslip coated with anti-CD3 for 5 min prior to fixation. pLck^{Y394} is shown in red and phalloidin in green.

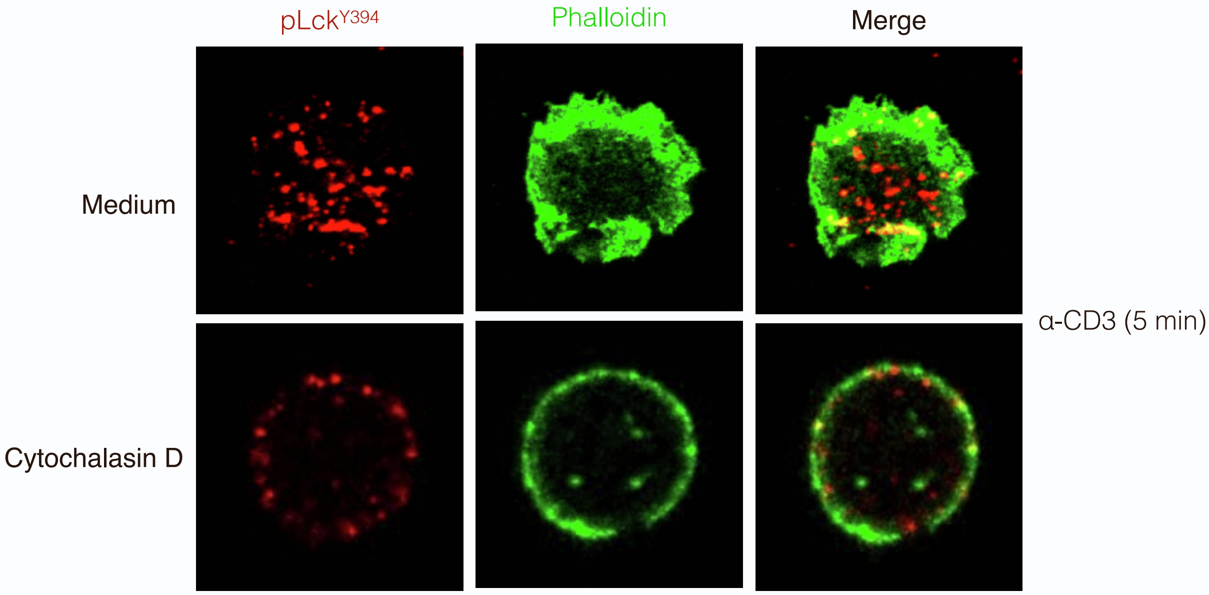

Figure. S4 Actin polymerization is required for the formation of microclusters
Jurkat WT cells treated with or without cytochalasin D were dropped onto a coverslip coated with anti-CD3 for 5 min prior to fixation. pLck^{Y394} is shown in red and phalloidin in green.
